# Effect of Healing Agents on Crack Healing of Asphalt and Asphalt Mortar

**DOI:** 10.3390/ma11081373

**Published:** 2018-08-07

**Authors:** Changluan Pan, Ping Tang, Martin Riara, Liantong Mo, Mingliang Li, Meng Guo

**Affiliations:** 1 State Key Lab of Silicate Materials for Architectures, Wuhan University of Technology, Wuhan 430070, China; panchangluan@whut.edu.cn (C.P.); tangping@whut.edu.cn (P.T.); m.m.riara@whut.edu.cn (M.R.); 2 Department of Physics and Electronics, South Eastern Kenya University, 170-90200 Kitui, Kenya; 3 Research Institute of Highway Ministry of Transport, Beijing 100088, China; ml.li@rioh.cn; 4 College of Architecture and Civil Engineering, Beijing University of Technology, Beijing 100124, China; gm@bjut.edu.cn

**Keywords:** asphalt, asphalt mortar, healing agents, crack healing, healing model

## Abstract

This study investigated the effect of seven healing agents on crack healing ability of long-term aged asphalt and its mortar. Different healing agents including sunflower oil, aromatic oil, bitumen emulsion, and maltene-based emulsions were used. The crack healing of asphalt made use of two asphalt disk samples and healing was evaluated using direct tensile tests. For asphalt mortar, notched semi-circular samples were used. Test results indicated that the crack healing of asphalt and its mortar depended strongly on the type of healing agent. In general, asphalt healed faster than its mortar. Asphalt healing could be well improved by using oil agents, while asphalt mortar could be well healed with maltene-based emulsions. The crack healing of asphalt mortar developed rapidly followed by a steady state of increase. Initial crack healing using healing agents could be contributed by the diffusion and softening effects, which resulted in low strength recovery. Long term healing could lead to the bonding reconstitution in the cracks, which were decisive for the final strength gain. The promising healing agent should be able to achieve maximum strength recovery to resist cracking as well as a sufficient re-healing ability to deal with crack opening and closing.

## 1. Introduction

Asphalt mixture usually consists of coarse aggregate, fine sand, filler, and bitumen. Asphalt pavements paved with hot asphalt mixture are safe, economic, and durable. Their design life is about 16 years for highways and freeways in China [1]. During the long period of service life, fatigue cracks, thermal cracks, and reflective cracks occur in asphalt pavements due to traffic loads and environmental conditions [2,3,4]. Cracks give easy access to water and moisture permeation in asphalt concrete, as well as pavement structure. As a result, water-induced damage could happen under the dynamic water pressure and pumping effect of repeated wheel loadings [5]. The premature distresses including stripping bleeding and pothole significantly affects the driving safety and comfort, as well as the service life of asphalt pavement. It is, thus, necessary to fill and seal the cracks in due course to prevent progressive water damage.

Bituminous materials have self-healing property, which is helpful for crack healing to recover stiffness, extend fatigue life and regain strength [6,7,8,9]. It was reported that induction heating and embedding microcapsules containing rejuvenator agents could promote crack healing [10,11,12,13,14,15,16,17]. Magnetic induction heating and microwave heating technology could heat bituminous materials and heal cracks [18,19,20,21,22,23]. Asphalt mixtures containing microcapsules were found to promote crack healing by release the rejuvenator agents into the cracks [24,25,26,27]. These technologies are effective in healing micro-cracks, while for macro-cracks, additional materials are needed to fill in the gap and promote crack healing [28,29].

Asphalt mortar is a composite material consisting of fine aggregates, fillers, and bitumen. It is an important binding material in asphalt concrete to hold coarse aggregates together and form the skeleton for loading support. Since asphalt mortar is rich of bitumen, it was found to significantly affect the performance of recycled asphalt mixtures [30]. Asphalt, asphalt mortar, and asphalt mixtures all have an important role on the crack resistance and healing of asphalt pavements. Some studies indicated that the self-healing properties of bituminous materials reduced at the direction of scale from bitumen, asphalt mastic, asphalt mortars, and asphalt mixtures. The addition of filler, fine sand, and coarse aggregates could significantly decrease the self-healing ability of bituminous materials [6,7,8,9]. It was found that the healing strongly depends on the crack size. Cracks at the meso scale and macro scale might hardly heal completely. It becomes necessary to introduce heat or healing agents to promote the healing of open cracks.

This study aims at a better understanding of the crack healing ability of asphalt and asphalt mortar containing healing agents. Different healing agents including sunflower oil, aromatic oil, bitumen emulsion, and maltene-based emulsion were used to promote the crack healing. The healing agents were applied on the fractured surfaces of long-term aged asphalt sample, as well as its mortar samples. The crack healing of asphalt samples was tested after healing for 1 day, 2 days, and 4 days by means of the strength recovery. For the mortar healing, longer healing time was considered for 1–60 days to improve healing. Mortar specimens were tested and the recovery of stiffness, strength, and fracture energy was used as healing indicators. Effects of various agents on crack healing were investigated and a model was proposed to predict the development of mortar healing over time.

## 2. Materials and Methods

### 2.1. Materials

Long-term aged asphalt and asphalt mortar which contained basalt aggregate, limestone mineral filler and SBS (Styrene Butadiene Styrene) modified asphalt was used in this study. Table 1 gives the aggregate gradation of the studied mortar. The gradation was determined from the fine fraction (smaller than 4.7 mm sieve size) of AC-13 mixture gradation specified in Chinese Standard of Highway Asphalt Pavement Design, JTG D20-2017 [1]. An SBS-modified asphalt binder that was commonly used in the surface wearing course was employed. Its main properties are as follows: penetration of 73 mm, ductility of 52.1 at 5 °C, and softening point of 68 °C. Long-term aged asphalt was obtained after subjected to RTFOT (Rolling Thin Film Oven Test) aging and PAV (Pressure Aging Vessel) aging according ASTM D6521-18 [31]. The used basalt aggregate had a Los Angeles abrasion value of 7.8%, a crushed stone value of 12.0%, a flakiness and elongation index of 8.5%, and a specific gravity of 2.961 g/cm^3^. Limestone mineral filler with a density of 2.83 g/cm^3^ and chemical composition of 51.8% CaO, 3.49% SiO_2_ and 1.29% Al_2_O_3_ was used. The asphalt content of asphalt mortar was determined by 9.2% by taking into account the optimum asphalt content of 4.7% for AC-13 asphalt mixture and the percentage of fine aggregate in the total mixed aggregate.

Seven different healing agents were used in this study and their origins are briefly described below. These healing agents used could be classified into sunflower oil, aromatic oil, bitumen emulsion, and maltene-based emulsion. The use of renewable bio-oil as a modifier for petroleum asphalt has recently been getting more attention [32,33,34]. Sunflower oil was, thus, to represent a renewable bio-oil as the healing agent. Aromatic oil was chosen because it is part of bitumen’s composition and was proven to have a significant effect on asphalt rejuvenation [35,36]. The content of aromatic hydrocarbon of the used aromatic oil employed was 78% according to ASTM D2140 [37]. The smoke point of sunflower cooking oil was 227 °C, while the flash point was 315 °C.

In order to compare the crack healing ability between these two types of oil and their emulsions, sunflower oil and aromatic oil were emulsified by using 60% oil, 2.5% cationic emulsifier, 0.3% stabilizer, and 37.2% water. The solution was controlled at 60–70 °C, while the oil was heated up to 110–120 °C. The oil emulsion was prepared by a high shear colloid mill for 2 min in the laboratory.

Two maltene-based emulsions, named HA-2, and HA-3 were commercial pavement maintenance agents. Both of them were cationic emulsions with a high content of aromatics. For the purpose of comparison, one base asphalt emulsion, named BBE was used. The related technical requirements of the used BBE meet with those for PC-1 base asphalt emulsion according to Technical Specification for Construction of Highway Asphalt Pavements, JTG F40-2004 [38].

In total, seven healing agents were used in this study, which are listed as follows: sunflower oil (KY), sunflower oil emulsion (KR), aromatic oil (FY), aromatic oil emulsion (FR), two commercial maltene-based emulsions (HA-2 and HA-3), and base asphalt emulsion (BBE). Table 2 gives the test results on the measured viscosity and the four components (saturates, aromatics, resins, and asphaltenes) determined by means of thin-layer chromatography (TLC) interfaced with flame ionization detection (FID) (IATROSCAN MK-6, LSI Medience Corporate, Tokyo, Japan) [39]. The particle size of the healing emulsions including KR, FR, HA-2, HA-3, and BBE agents was observed by an XSP-16A biological microscope produced by Nanjing Jiangnan Yongxin Optical Co., Ltd. (Nanjing, China) Almost all of particles are less than 10 μm and more than 80% of them were less than 5 μm.

### 2.2. Sample Fabrication

**Figure 1:**
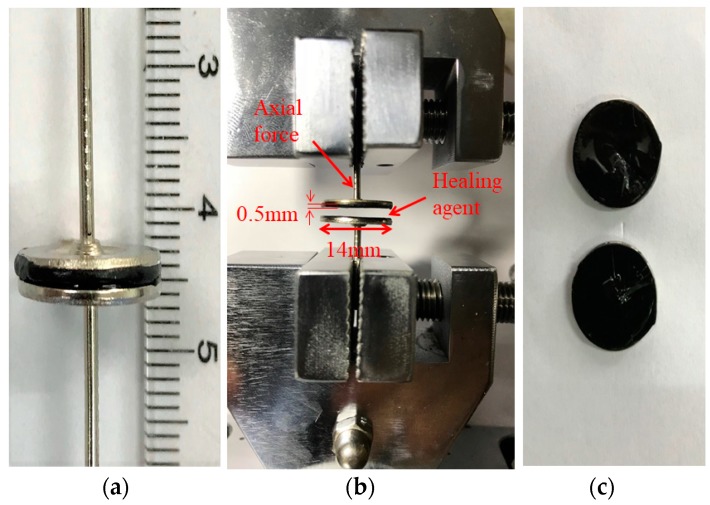
Asphalt sample (**a**), test setup (**b**), and the fractured surfaces (**c**).

The asphalt sample was prepared by using two pieces of asphalt disks as indicated by Figure 1. To prepare the asphalt disk, a small drop of asphalt was placed on the surface of a round nail 14 mm in diameter and pressed to obtain a thick asphalt film around 0.4–0.6 mm. Two nails coated with asphalt films were put together with or without applying healing agents on the surface of asphalt disks. After applying the healing agent, the two disks were adhered together vertically and compressed by the weight of the top nail. In order to determine the asphalt tensile strength, a similar asphalt sample, but with one asphalt disk of 1 mm thickness to adhere two nails was prepared. The tensile strength obtained on this asphalt sample that only consists of one asphalt disk is defined as the original strength.

**Figure 2:**
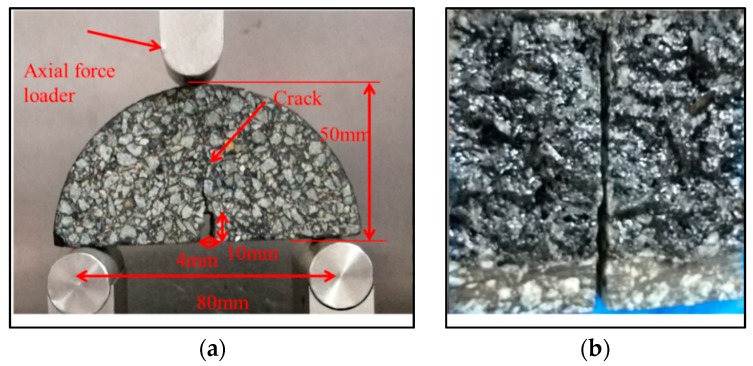
Semi-circular bending test setup (**a**) for mortar sample and the fractured surfaces (**b**) after testing.

Figure 2 presents an illustration for the semi-circular bending test setup on the asphalt mortar sample. Semi-circular bending test (SCB) specimens were prepared by taking into account the effect of long-term aging. Long-term aging was simulated by placing loose asphalt mortar in an oven at 100 °C for 96 h [40,41]. Marshall specimens of 100 mm diameter and 63 mm height were prepared using 75 blows per face. Notched SCB samples of 100 mm diameter with a notch 4 mm thick and 10 mm deep at the midpoint were carefully sawn from Marshall specimens according to AASHTO TP105 [42].

### 2.3. Test Procedures

A tensile testing machine (ZQ-990A, Zhiqu Precision Instruments Co., Ltd., Dongguan, China) was used to carry out the asphalt sample test. The blank asphalt sample was referred to the one without applying healing agents. It was prepared by adhering the two asphalt disks directly together. The other samples were prepared by applying various healing agents on one surface of each pair of asphalt disks. The healing agent was applied by using a soft brush and the application rate was carefully controlled at 0.2–0.3 kg/m^2^. After that, the pair of asphalt disks were adhered together and healed for different periods of time (1, 2, and 4 days) at room temperature around 25 °C. After reaching the desired healing time, a direct tensile test was performed at −10 °C with a load rate of 50 mm/min. Before starting the test, the samples were kept in an environmental cabinet at −10 °C for 1 h to ensure the test samples reached the required test temperature. The effects of 1, 2, and 4 days on asphalt healing were investigated. In this test, three replicate samples in each group were conducted, and the mean value of test result was used for data analysis. Similarly, the asphalt sample that only consists of one asphalt disk was subjected to direct tensile testing under the same test conditions. The obtained results was defined as the original strength, which was used as the unique reference to determine the healing index from the asphalt samples consisting a pair of asphalt disks.

A universal testing machine (UTM-25, IPC Global, Victoria, Australia) was used to artificially make asphalt mortar cracks. This test was divided into three steps:

- Initial fracture of the SCB samples: The test was conducted at −10 °C with a load rate of 0.5 mm/min. Before starting the test, the samples were kept in an environmental cabinet at −10 °C for 4 h to ensure the test specimens reach the required test temperature. Each sample was loaded until completely broken. The fractured sample was moved to room temperature for at least 2 h to raise the temperature of the sample to around 25 °C.
- Application of the healing agents: A soft brush was used to apply the healing agents on the cracked surfaces at a spreading rate ranging from 0.3 to 0.7 kg/cm^3^. This rate was appropriate to ensure that the healing agents were fully wetted in the cracked surface without excessive outflow. After the healing agents were applied, the samples were carefully placed together and excess healing agents were wiped. Then they were placed vertically at room temperature.
- After 1 day of healing, the samples were tested again. In order to avoid moisture attachment on the cracked surfaces, which would affect the healing of the samples, when they were completely broken, they were put at room temperature for 2 h. The same fractured samples were then put together carefully without the re-application of the healing agents. After 2 days of healing, the same specimen was tested. By the fracture and re-heal method, the effect of 4, 8, 30, and 60 days on crack healing was evaluated. In this test, at least three replicate samples in each group were conducted, and the mean value of test result was used for data analysis.

### 2.4. Healing Indicators

The healing of asphalt materials is usually evaluated by the recovery of the material’s mechanical properties, such as stiffness, tensile strength, fatigue life, and dissipative energy [7,11,20]. The commonly used healing index is the ratio of the material strength after healing to the original strength. In this case, a higher ratio indicates a better healing performance.

**Figure 3:**
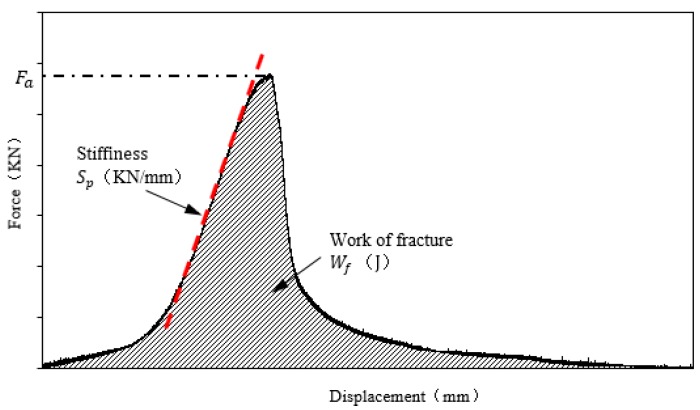
Illustration of determination on peak strength, stiffness, and fracture energy based on the load-displacement curve.

In this study, the healing index obtained from the recovery of strength was used to measure the healing ability of asphalt. In order to get more insight into the crack resistance after healing, healing indices including the recovery of strength, stiffness, and fracture energy as indicated in Figure 3 were used to explore the crack healing of asphalt mortar. In this paper, the three healing indices mentioned above were determined according to AASHTO TP105 [42] and are shown in Equations (1)–(6).

Strength healing index (FI) is the strength recovery ratio calculated by:

(1) $$\;\mathrm{FI}=\frac{F_{\mathrm{ah}}}{F_{\mathrm{ai}}}\times 100\%\;$$

where the subscripts “i” and “h” indicates the peak force *F*_a_ tested initially and after the healing, respectively.

Stiffness Index (SI) is the stiffness recovery ratio determined by:

(2) $$\;\mathrm{SI}=\frac{S_{\mathrm{ph}}}{S_{\mathrm{pi}}}\times 100\%\;$$

The subscripts “i” and “h” presents the mortar stiffness *S*_p_ tested initially and after the healing, respectively.

Fracture Energy Index (EI) is the fracture energy ratio:

(3) $$\;\mathrm{EI}=\frac{E_{h}}{E_{i}}\times 100\%\;$$

(4) $$\;E_{i/h}=\frac{W_{f}}{A_{\mathrm{lig}}}\;$$

where “i” and “h” are fracture energy tested initially and after the healing, respectively. Ligament area (*A*_lig_) and the work of fracture (*W*_f_) are determined by Equations (5) and (6), respectively.

(5) $$\;A_{\mathrm{lig}}=\left(r-a\right)t\;$$

where *r*, *a* and *t* are sample radius, notch length and specimen thickness, respectively.

(6) $$\;W_{f}=\sum_{i}^{n}\frac{1}{2}\left(F_{i+1}+F_{i}\right)\left(d_{i+1}-d_{i}\right)\;$$

where *F_i_* and *F_i_*_+1_ present applied load at *i* and *i* + 1 load step application, respectively, and *d_i_* and *d_i_*_+1_ are the displacement at the *i*-th and *i* + 1 position.

## 3. Results and Discussion

### 3.1. Effect of Healing Agents on Asphalt

**Figure 4:**
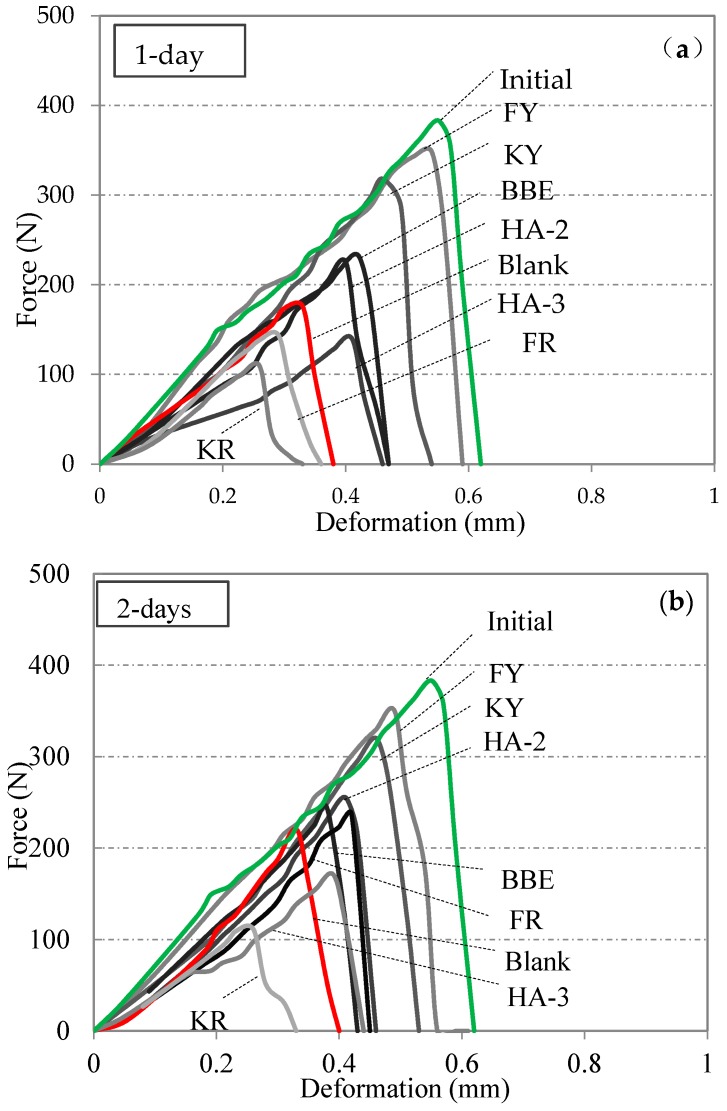

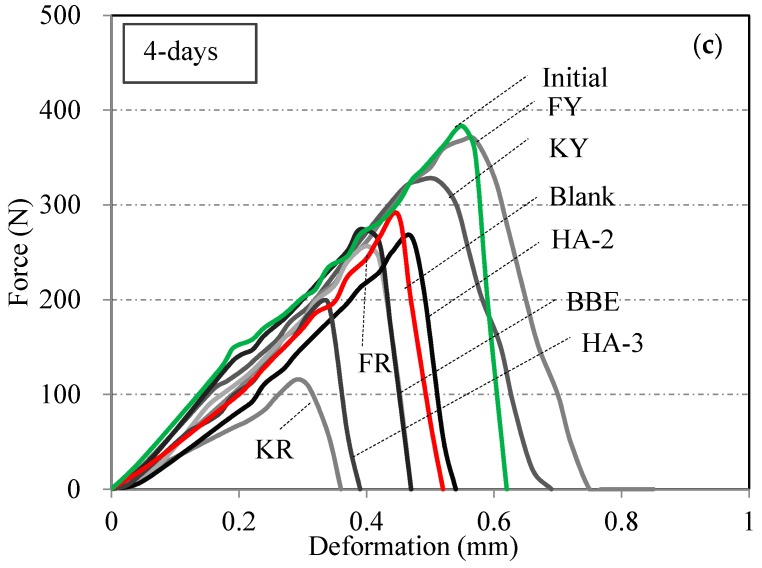
Direct tensile testing results on asphalt samples treated with various healing agents and healed for 1 day (**a**), 2 days (**b**), and 4 days (**c**).

**Figure 5:**
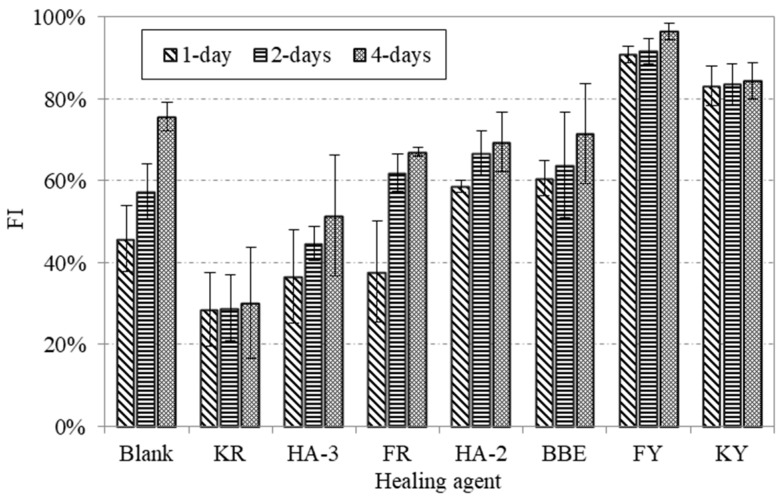
Healing index of strength recovery (FI) for various healing agents determined by taking into account the original bitumen tensile strength of 383 N.

Figure 4 shows the results obtained from direct tensile testing on blank asphalt samples and those treated with various healing agents. Data obtained from the one-disk asphalt sample were also added for the purpose of reference. Different healing periods of time including 1, 2, and 4 days were involved. Based the force-displacement curves, the peak forces of healed asphalt samples were determined. Furthermore, the original bitumen tensile strength obtained from the one-disk asphalt sample was determined as 383 N. By comparing the peak forces with the original bitumen tensile strength, the healing index of strength recovery were presented in Figure 5.

As indicated in Figure 4, different healing agents displayed distinct behavior on the force-displacement curves. The healing effect of each agent was well distinguished from the peak force. For better interpretation of the healing ability of asphalt, the healing index (FI) shown in Figure 5 will be used for the following discussion. As can be seen, the blank asphalt sample had an obvious healing ability. The healing index was 46% for one day of healing and extended healing time could result in an increased healing index. When the healing time reached 4 days, up to 76% strength recovery could be obtained. Among the seven healing agents, the category of non-emulsion agents, for example, FY and KY showed the highest values of healing index. The emulsion type of the healing agents tended to show a complex healing behavior due to the presence of water. It was expected that the evaporation of water between two asphalt disks significantly affected the gain of strength. This was well observed by comparing KY with KR. Similar results could also be seen between FY and FR. The application of traditional bitumen emulsion (BBE) could improve the crack healing, especially at the initial phase when compared with the blank asphalt sample. Two maltene-based emulsions (HA-2 and HA-3) did not show a consistent trend on the improvement of crack healing. HA-2 had a higher healing index before 2 days of healing compared to the blank asphalt sample, while a lower value was found for HA-3. This indicated that HA-3 and KR did not promote the healing of asphalt during 4 days of healing. The data presented in Figure 5 shows that the asphalt could have a considerable healing ability even after long-term aging. Oil healing agents could really promote the healing of asphalt. This is attributed to the dissolution and wetting effects of this type of agent. Some emulsion agents showed limited effect on healing because the presence of water between the two asphalt disks evaporated slowly and, thus, impeded the emulsion breaking. This resulted in low strength gain, especially at the initial phase.

### 3.2. Appearance of Fractured Surface for Asphalt

**Figure 6:**
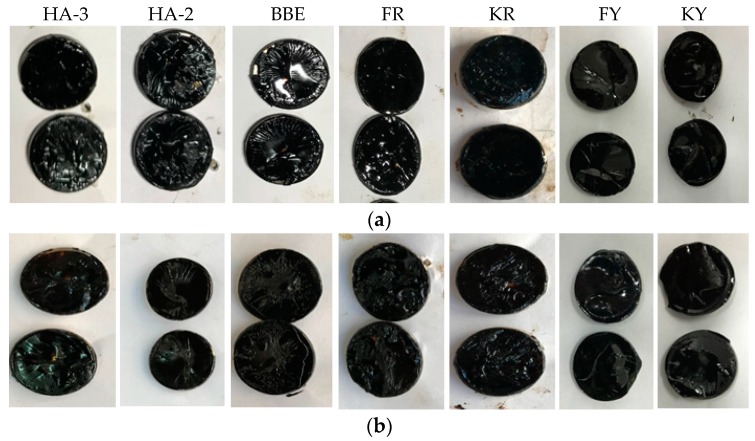
Fracture surfaces for various asphalt samples after (**a**) 1 day and (**b**) 4 days of healing.

Figure 6 shows the fractured surfaces of asphalt samples healed by various healing agents. It should be noted that the direct tensile testing was performed at −10 °C. At such a low temperature, asphalt, especially after long term aging, tended to have a brittle failure. When the pair of asphalt disks was adhered together, the crack interface could be the weak link for fracture if the crack was not completely healed. For this reason, the observation of the fracture surfaces could be very useful for evaluation of failure mode, as well as the diffusion of the healing agents. Three failure modes could be expected: failure within the crack interface, failure through the asphalt disks, and adhesive failure between asphalt disk and the nail. After inspection of all the fractured samples, it was found that all of the nails were covered with black asphalt binder and the adhesive failure between the asphalt disk and the nail was hardly seen. This indicated that the adhesive strength between the asphalt disk and the nail was much higher than the cohesive strength of the asphalt, as well as the crack healing strength. Among the seven healing agents, sunflower oil (KY) and aromatic oil (FY) provided strong healing effects for asphalt cracks. For this reason, the fracture surfaces tended to occur within the asphalt disks, but not the crack surfaces. However, sunflower oil emulsion (KR) exhibited the lowest healing effect. Therefore, the failure was concentrated on the crack interface between two asphalt disks. This was proven by the observation of sticky residue on both surfaces of the fractured sample. When the healing time was increased to 4 days, the same failure mode happened, but drier and harder fracture surfaces were observed. This indicated that the healing agent was gradually diffused in the asphalt disks and, thus, led to increased healing strength. When the healing index was higher than 70%, the failure was likely changed from the crack interface to the asphalt disks, or a mixed failure of both could be observed. The above discussion indicates that some of the used healing agents could really improve the crack healing of asphalt and prevent crack failure. The strength gain depended on the interaction between the asphalt and the used healing agents. Extended healing time, thus, improved the healing strength. The failure mode and the hardness of the fracture surfaces could be an indicator for the interaction between asphalt and the healing agent.

### 3.3. Healing of Asphalt Mortar

Table 3 and Table 4 present a summary of test results obtained on the strength, stiffness, and fracture energy of asphalt mortar treated with and without healing agents. It should be noted that the initial strength, stiffness, and fracture energy stand for the original mechanical properties. When the mortar specimens were cracked and healed without treating with healing agents, the recovery of strength, stiffness, and fracture energy was very low. It indicated that the self-healing ability of mortar samples was insufficient to repair cracks at room temperature. On the contrary, mortar samples treated with healing agents showed an obvious increase in these mechanical parameters, which implied that healing agents were effective to promote mortar crack healing.

It can also be seen that healing time played an important role in the healing of asphalt binder. As expected, extended healing time could promote the development of crack healing. Among the recovery of strength, stiffness and facture energy, strength, and fracture energy showed a clear increase trend as the healing time increased, while stiffness did not show a particular change tendency. This, therefore, indicated that stiffness could not be employed as a healing index of crack repair of asphalt mortar. Compared with the recovery of fracture energy after treating with healing agents, the strength gain increased rapidly with healing time and, thus, may well distinguish the development of crack healing from various healing agents. Considering that fracture energy represents the crack resistance and propagation, both of strength and fracture energy were used as healing indicators to evaluate the crack healing performance of asphalt mortar treated with various agents.

### 3.4. Appearance of Fractured Surface for Asphalt Mortar

**Figure 7:**
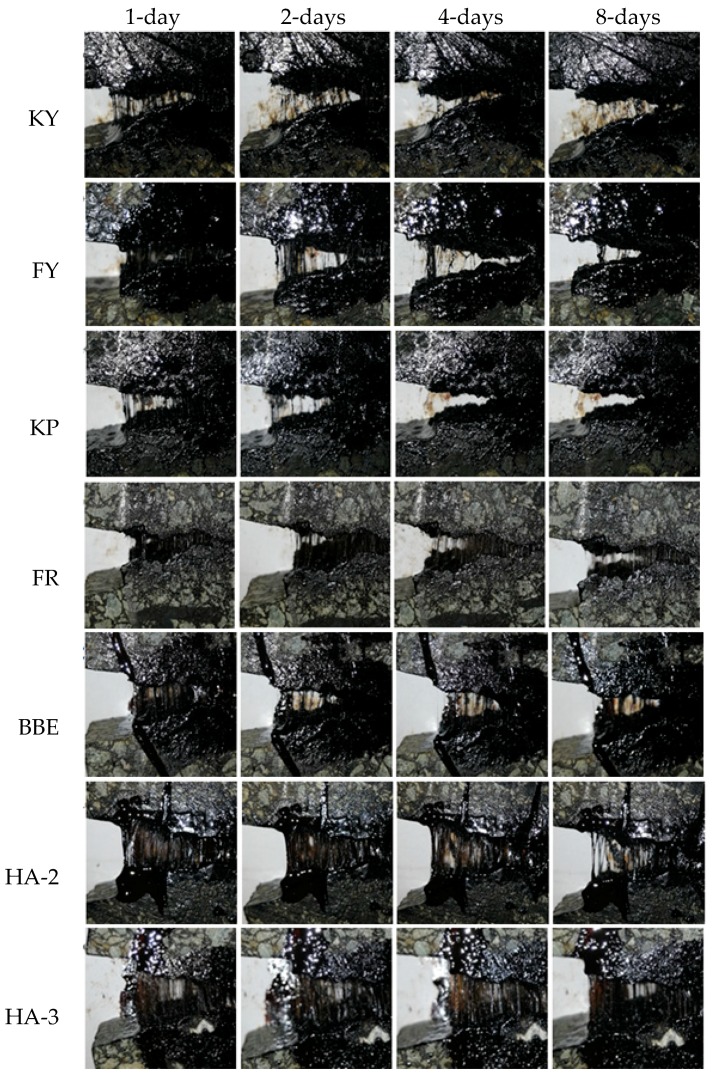
Appearance of the cracked surfaces for healed asphalt mortar after different healing times.

Figure 7 shows the appearance of the cracked zones for mortar specimens after 1, 2, 4, and 8 days healing. These pictures were taken after the applied load reached the peak value during testing. As can be seen, the cracked zones treated with sunflower oil (KY) and aromatic oil (FY) showed filaments in the initial days. However, these filaments tended to disappear after longer healing time. This similar phenomenon was also observed on the pictures of sunflower oil emulsions (KR) and aromatic oil emulsion (FR). The water in their two emulsions tended to reduce the crack healing when compared with the un-emulsified oil.

The quantitative data showed in Table 3 and Table 4 indicated that BBE, HA-2 and HA-3 had relative better healing performance. This is in agreement with the qualitative data in Figure 7 indicated by large amount of thin filaments which could be seen even after 8 days healing. In addition, the fracture of healed mortar samples was caused by cohesive failure. This indicated that the strength recovery of mortar crack really depends on the interaction between mortar and healing agent. According to Guo’s study, the four components (saturates, aromatics, resins, and asphaltenes) of bitumen had a great influence on diffusion, adhesion, and cohesion work [43]. High diffusion speed of low molecular weight saturates and aromatics helped to activate and soften the asphalt binder on the crack surfaces and thus promote healing. High adhesion work of aromatics and asphaltenes could prevent interfacial adhesive failure. Cohesion strength would benefit from resins and asphaltenes. The results showed in Table 2 indicated that aromatics rich agents could really improve the crack healing. However, the existence of aromatics was likely to act as softening additive for the cracks. The softened asphalt binder increased self-healing ability, but reduced the cohesive strength. This made the cracks hardly recover to their original strength of the mortar. Combined Figure 7 and Table 2, it is found that healing agents should have high diffusion ability and softening effect to mobilize self-healing, while they should be able to reconstitute the chemical and physical properties of the binder phase between two crack surfaces and recover strength.

### 3.5. Effect of Healing Agents on Fracture Energy Healing Index

**Figure 8:**
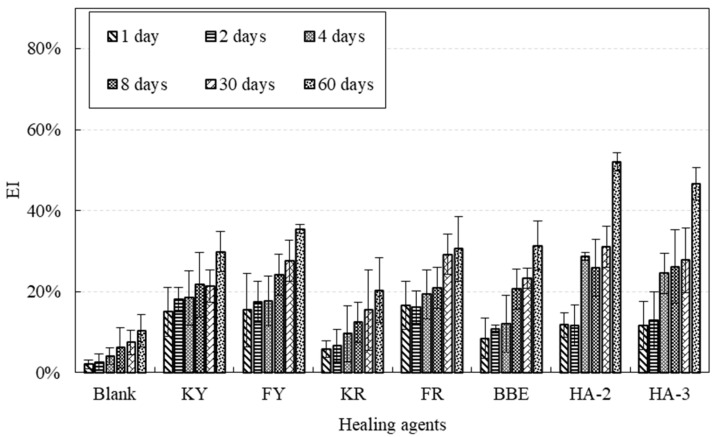
EI of asphalt mortar treated with healing agents after different healing times.

Figure 8 shows the test results of fracture energy healing index (EI) of asphalt mortar treated with various healing agents. The EI value was determined by the ratio of the fracture energy after crack healing compared to the original one. The results indicated the crack healing really depended on the type of healing agent. It also demonstrated that increased healing time led to increase in EI. For instance, mortar samples healed with the oil agents (KY and FY) had an increase in EI from 15.01% to 29.86% and 15.44% to 35.48% when healing time increased from 1 to 60 days. However, the corresponding samples healed using their emulsion KR and FR had a lower EI ranging from 5.84% to 20.36% and 16.59% to 30.61%, respectively. An obvious increase in EI can be seen on mortar samples treated with HA-2 and HA-3. During the initial 8 days of healing, the difference in EI could be considered relatively small. However, the results obtained on 60 days of healing were distinct. The reasons for such a difference could be explained by the short-term healing and long-term healing. For the short-term healing, diffusion and softening effect are primary, which just motivates the self-healing ability of the crack surfaces. As a result, the strength recovery and crack resistance are relatively low. During long-term healing of 60 days, the reconstitution of the binder could lead to improved adhesion and cohesion, thus, recovering strength to a higher level.

### 3.6. Effect of Healing Agents on Strength Healing Index

**Figure 9:**
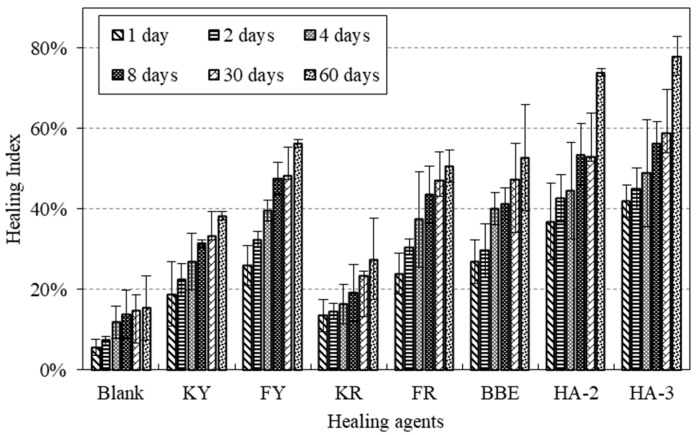
FI of asphalt mortar treated with/without healing agents after different healing times.

Figure 9 presents the test results of strength healing index (FI) of various healing agents. It should be noted that FI was calculated by ratio of the strength after healing compared to the original strength. As indicated, the strength recovery for mortar samples without applying healing agents was very low. For instance, after 1 day healing, the FI was 5.59%, and it increased to 11.92%, 13.84%, 14.72% and 15.34% for 4, 8, 30, and 60 days, respectively. This showed that the self-healing process reached a maximum value and further healing was limited. Therefore, it is necessary to improve the self-healing ability of asphalt materials to achieve a higher degree of crack healing.

Among the seven healing agents, sunflower oil (KY) and its emulsion showed the lowest degree of crack healing, followed by FR, FY, BBE, and HA-2; HA-3 seemed to have the highest healing effect. It indicated that the application of sunflower oil had a limited strength recovery lower than 40%. In general, the application of aromatic oil had a better effect than traditional base bitumen emulsion. Mortar samples healed with the maltene-based emulsion healing agents (HA-2 and HA-3) had an excellent healing effect. For example, the healing index could be up to 50% after eight days of healing.

The effect of healing time on FI was similar to those of EI as indicated in Figure 8. In general, extended healing time resulted in higher FI value. After one day of healing, sunflower oil (KY and KR) had a FI value lower than 20%; BBE and aromatic oil (FY and FR) could increase to 30%, while maltene-based emulsion healing agents (HA-2 and HA-3) could be close to 40%. Extended healing time to 60 days, the FI values of the mentioned four categories could reach around 40%, 55%, 60%, and 75%, respectively. The above data clearly showed that the difference between the initial healing effect and the long-term healing. As mentioned before, the low strength recovery in the initial phase may be due to the diffusion and softening effect, while the obvious strength gain was obtained from the reconstitution of the binder phase including healing agent and original in-mortar bitumen after long-time of healing.

### 3.7. Modelling of Healing Development

According to above results, the healing time plays a significant role in crack healing of asphalt mortar. To find out the relationship between EI and healing time, Equation (7) was proposed to describe the healing process of the mortar in the entire period of time.

(7) $$\;\mathrm{HI}\;\left(t\right)=A-B\;exp\left(\frac{-t}{t_{o}}\right)\;$$

where *A* is the ultimate EI, *B* is the degree of healing development, (*A* − *B*) is the initial EI, and *t*_0_ is the shift point. Below *t*_0_, the healing rate develops rapidly and above *t*_0_, the healing rate tends to increase steadily.

**Figure 10:**
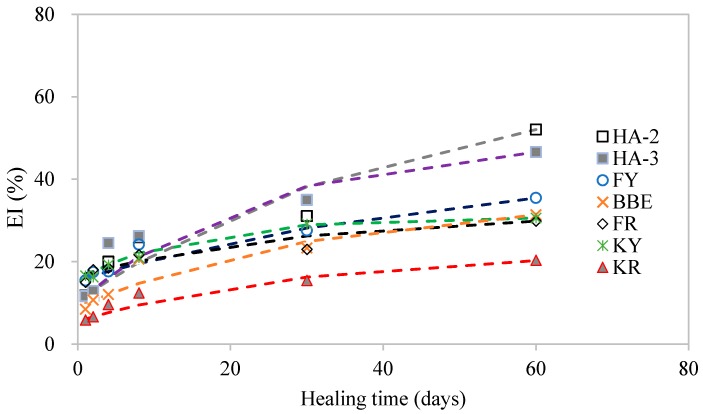
Comparison of model prediction and test results of EI development over healing time.

The fitting parameters using Equation (7) for various healing agents are shown in Table 5. Good fitting with high *R*^2^ values indicated that Equation (7) was appropriate for modelling the healing development based on fracture energy. Figure 10 presents a comparison of model prediction and test results of EI development over healing time. As indicated, the model predicted well the EI development over healing time. As can be seen in Table 5, the initial EI indicated by the fitting value of *A* − *B* varied from 5% to 16%. This implied that the initial recovery of EI was relatively low. The ultimate EI indicated by the value of A distinguish well the crack healing performance of various healing agents. Sunflower oil (KR and KY) tended to have poor performance while maltene-based emulsion (HA-2 and HA-3) behaved excellently.

For the relation between FI and healing time, a healing model in Equation (8) developed by Wool and O’Connor [44] was used:

(8) $$\;\mathrm{FI}\left(T,t\right)={\mathrm{FI}}_{0}+D\left(T\right)t^{0.25}\;$$

where FI(T,t) are the FI of healing agents at the temperature *T* and time *t* respectively; *T* is healing temperature in degree K; *t* is healing time (s); ${\mathrm{FI}}_{0}$ are the initial strength healing index; *D*(*T*) indicates the strength gain rate due to the inter-diffusion of molecules between the crack surfaces at temperature *T*; *D*(*T*) is defined as the strength healing rate which can be explained by the Arrhenius law of diffusion as defined in Equation (9):

(9) $$\;D\left(T\right)=D_{0}\exp\left(-\frac{E_{a}}{RT}\right)\;$$

where: $D_{0}$ is diffusion constant; $E_{a}$ represents the minimum energy required for healing; *R* is the universal gas constant (8.314 J mol^−1^ K^−1^).

In this study, only one healing temperature was involved. Thus, Equation (8) can be further simplified as:

(12) $$\;\mathrm{FI}\left(t\right)=FI_{0}+Dt^{0.25}\;$$

where model parameters $FI_{0}$ and *D* can be obtained by fitting the test data.

**Figure 11:**
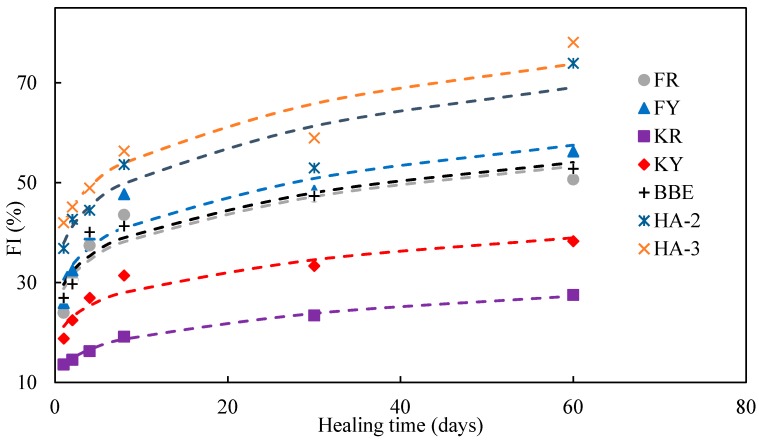
Correlation of healing time and FI.

**Figure 12:**
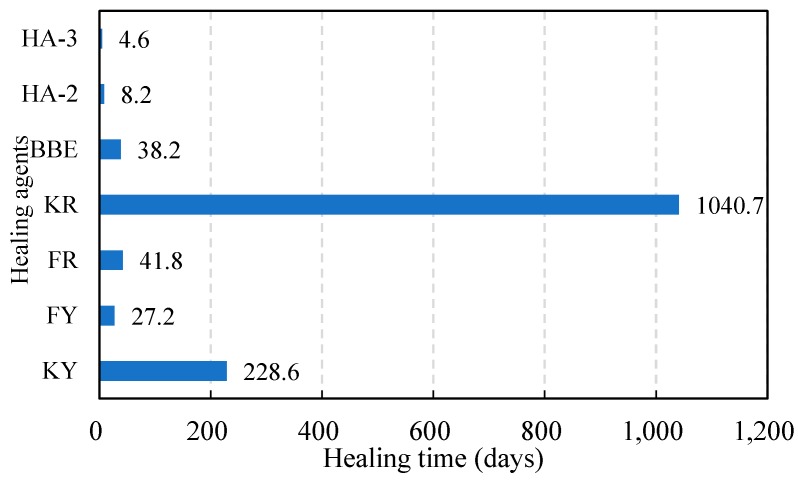
Healing time required for the FI to reach 50%.

Table 6 gives the model fitting results on FI development over time for various healing agents. The model parameters $FI_{0}$ and $D_{2}$ were determined by least squares regression. The correlation coefficient for each healing agent was larger than 0.87. A comparison analysis between the test and modeled FI results is presented in Figure 11. As indicated, the proposed model could well predict the development of FI over time. The FI increased rapidly at the beginning and then came to a steady state. Sunflower oil had lower value of $FI_{0}$ and $D_{2}$, which indicated a lower initial strength gain and healing rate compared with other healing agents. The highest initial strength gain and healing rate was found for HA-3. This further proved a good healing performance of HA-3. With the healing modeling results listed in Table 6, the healing time required for the healing agents to recover 50% of its original strength could be calculated as shown in Figure 12. Sunflower oil (KY and KR) had longest healing times which were 228.6 and 1040.7 days. The time extrapolation based on the model to more than 1000 days were difficult to validate due to the time consuming. This showed the limitation of the model. BBE and aromatic oil (FY and FR) needed 38.2, 27.2, and 41.8 days, respectively. The minimum healing time for maltene-based emulsions (HA-3 and HA-2) to achieve half of healing was 4.6 and 8.2 days respectively, which showed the shortest healing time. The analysis above indicated that sunflower oil lacked strength improvement ability and, thus, was not a good option for crack healing. BBE and aromatic oil exhibited a similar behavior at both initial strength gain and healing rate. Maltene-based emulsions showed the best healing performance. The highest healing rate also indicated a positive reconstitution effect during long-term healing.

## 4. Conclusions

Seven healing agents, including sunflower oil (KY), sunflower oil emulsion (KR), aromatic oil (FY), aromatic oil emulsion (FR), two commercial maltene-based emulsions (HA-2 and HA-3), and base asphalt emulsion (BBE) were used to heal cracks of fractured long-term aged asphalt and semi-circular samples of long-term aged AC-13 asphalt mortars. The healing ability of these healing agents was evaluated using the strength and fracture energy indicators. In addition, a healing model was introduced to explain the crack healing development over healing time. Based on the data obtained and the analysis presented in this paper, the following conclusions could be drawn:

- Asphalt could have high self-healing ability even after subjecting to long-term aging. The crack healing of asphalt can be well improved by using oil agents but not emulsion agents.
- The healing ability for cracked asphalt mortar without any healing agents was low. The application of healing agent could significantly promote the crack healing. The strength recovery could well distinguish the healing ability of mortar cracks.
- The crack healing of asphalt and its mortar strongly depended on the type of agent used and the dependency was not in agreement with each other, indicating the difference of healing mechanisms of these two materials. Asphalt was much easier to heal compared with its mortar. The diffusion, softening, and reconstitution effects of the healing agent were important for crack healing.
- The healing development of mortar crack can be divided into the initial phase of rapid increase and a steady state. The diffusion and softening effects due to the application of the healing agent contributed to the initial low strength recovery. Long-term healing benefited the bonding reconstitution in the cracks and, thus, improved the final strength.
- Results on the fracture surfaces indicated that the behavior of crack healing was similar to asphalt rejuvenation. Therefore, it could provide a way to optimize the components of healing agent to achieve maximum strength recovery to resist crack, as well as sufficient re-healing ability to deal with crack opening and closing.

## Figures and Tables

**Table 1:** Aggregate gradations for the AC-13 mortar.

Sieve Size (mm)	Upper Limitation (%)	Lower Limitation (%)	Passing Percent (%)
4.75	100	100	100
2.36	100	48	66.8
1.18	76	30	50.2
0.6	56	20	32.8
0.3	40	14	22.5
0.15	30	10	14.5
0.075	16	8	9.6

**Table 2:** Chemical composition and viscosity of the healing agents.

Healing Agents	Chemical Composition (%)				Viscosity	
	Saturates	Aromatics	Resins	Asphaltenes	Residue at 60 °C (Pa s)	Emulsion at 25 °C (cp)
KY	1.5	86.3	10.0	2.2	0.5–5	–
FY	7.3	84.5	3.3	4.9	30–50	–
KR	1.5	86.3	10.0	2.2	0.5–5	30–50
FR	7.3	84.5	3.3	4.9	30–50	40–60
HA-2	18.1	48.6	22.2	11.1	140–160	50–70
HA-3	12.2	70.1	11.2	6.5	100–120	45–65
BBE	14.1	29.6	43.5	12.8	210–230	150–170

Note: KY = sunflower oil; KR = sunflower oil emulsion; FY = aromatic oil; FR = aromatic oil emulsion; HA-2 and HA-3 = two commercial agents; BBE = base asphalt emulsion.

**Table 3:** Test results of strength, stiffness, and fracture energy obtained from blank, sunflower oil-, and aromatic oil-treated mortar samples.

Healing Agents	Healing Time (days)	Strength (kN)	Stiffness (N/mm)	Fracture Energy (J/m^2^)
Blank (pure asphalt)	Initial	3.15	9.47	4.35
	1	0.18	0.80	0.09
	2	0.23	0.93	0.11
	4	0.38	2.87	0.18
	8	0.44	2.99	0.27
KY oil	Initial	3.27	9.34	3.90
	1	0.62	5.92	0.56
	2	0.74	6.39	0.67
	4	0.88	7.06	0.69
	8	1.03	6.87	0.81
FY oil	Initial	2.92	11.81	3.97
	1	0.76	4.83	0.56
	2	0.95	6.91	0.63
	4	1.16	7.58	0.70
	8	1.39	5.34	0.87
KR emulsion	Initial	3.41	8.90	4.55
	1	0.46	2.65	0.27
	2	0.50	2.99	0.30
	4	0.55	2.37	0.44
	8	0.65	3.08	0.57
FR emulsion	Initial	2.74	9.26	3.63
	1	0.66	3.11	0.60
	2	0.84	5.52	0.59
	4	1.02	7.43	0.70
	8	1.19	5.21	0.76

**Table 4:** Test results of strength, stiffness and fracture energy obtained from base asphalt emulsion and commercial maltene-based emulsion treated mortar samples.

Healing Agents	Healing Time (days)	Strength (kN)	Stiffness (N/mm)	Fracture Energy (J/m^2^)
BBE emulsion	Initial	3.20	9.19	4.21
	1	0.86	7.82	0.36
	2	0.95	7.30	0.45
	4	1.28	9.93	0.51
	8	1.32	5.85	0.87
HA-2 emulsion	Initial	2.99	6.67	3.86
	1	1.10	3.66	0.45
	2	1.28	2.94	0.45
	4	1.33	8.86	1.11
	8	1.60	8.63	1.00
HA-3 emulsion	Initial	3.16	8.85	4.20
	1	1.33	3.90	0.49
	2	1.43	8.87	0.54
	4	1.55	6.04	1.03
	8	1.78	7.45	1.10

**Table 5:** Fitting functions of EI for seven healing agents.

Healing Agents	*A*	*B*	*A* − *B*	*t*_0_	Correlation Coefficients
KR	22.7	17.1	5.5	30.4	0.94
KY	30.9	15.3	15.6	14.4	0.99
FR	32.0	15.4	16.6	30.3	0.89
FY	45.5	30.0	15.5	54.9	0.94
BBE	35.8	26.7	9.0	33.2	0.91
HA-2	66.0	55.5	10.5	43.4	0.92
HA-3	51.0	40.0	10.0	24.3	0.90

**Table 6:** Fitting functions of FI for seven healing agents.

Healing Agents	Fitting Function	Correlation Coefficients
KY	$\mathrm{FI}(t)=0.5808t^{0.25}+11.282$	*R*^2^ = 0.91
FY	$\mathrm{FI}(t)=0.8789t^{0.25}+15.597$	*R*^2^ = 0.87
FR	$\mathrm{FI}(t)=0.8016t^{0.25}+15.053$	*R*^2^ = 0.87
KR	$\mathrm{FI}(t)=0.4569t^{0.25}+5.508$	*R*^2^ = 0.99
BBE	$\mathrm{FI}(t)=0.7982t^{0.25}+15.980$	*R*^2^ = 0.91
HA-2	$\mathrm{FI}(t)=1.0192t^{0.25}+20.448$	*R*^2^ = 0.87
HA-3	$\mathrm{FI}(t)=1.0511t^{0.25}+23.653$	*R*^2^ = 0.92
